# How Various Drug Delivery Methods Could Aid in the Translation of Genome Prime Editing Technologies

**DOI:** 10.1155/2022/7301825

**Published:** 2022-02-21

**Authors:** Elena Ivanova

**Affiliations:** Nanotherapeutics and Stem Cell Engineering Lab, Department of Biomedical Engineering, Columbia University, 3960 Broadway, Columbia University Medical Center, New York, NY 10032, USA

## Abstract

Drug delivery systems can be engineered to enhance the localization of therapeutics in specific tissues in response to externally applied stimuli and/or local environmental changes. In recent decades, efforts to improve drug delivery techniques at both nano- and macroscale have led to a new era of therapeutic efficacy. Such technological advancements resulted in improved drug delivery systems regularly entering the clinical setting. However, these delivery innovations are unfortunately not always readily applied to newly developed technologies. One of these new and exciting technologies that has been overlooked by drug delivery scientists is prime editing. Prime editing is a novel genome editing technology that exhibits the plug-and-play capability of CRISPR/Cas9 editors while avoiding double-strand DNA breaks throughout the entire process. This article focuses on describing the potential advantages and disadvantages of selecting nanomedicine technologies along with prime editing capabilities for the delivery of cargo.

## 1. Introduction

The field of drug delivery has overcome many pharmaceutical hurdles. However, the majority of drug delivery research continues to focus on only a subset of pharmaceutical agents and diseases. A chief example of this “pharmaceutical neglect” is the lack of proposed delivery strategies for the new, high-interest field of genome prime editing. Since clustered regularly interspaced short palindromic repeats (CRISPR)-inspired prime editing technology has very recently been described [1], its publication has resulted in hyped reports from several media outlets, the founding of a start-up company, and the receipt of venture capital investments. In essence, this technology allows for flexible genome manipulation without double-strand DNA breaks observed in standard CRISPR/Cas9 systems. Despite the broad potential to create novel treatments for a plethora of genetic diseases, no proposals have yet been shared in the public domain for an effective nonviral delivery system for prime editing technologies. This article focuses on identifying the requirements for an effective prime editing delivery system, evaluation of advantages and disadvantages of current nanomedicine vehicles, and a proposal for which research areas should be pursued.

## 2. Prime Editing

**Figure 1:**
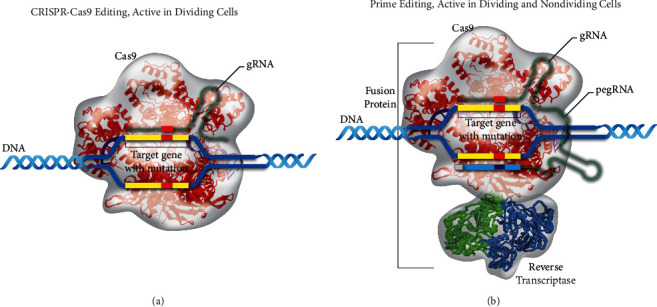
Two genome-editing approaches: (a) active CRISPR-Cas9 editing in dividing cells and (b) prime editing, active in dividing and nondividing cells.

Prime editing technologies have the potential to essentially revolutionize current genome-editing practices. Since the CRISPR/Cas9 system has been first utilized, adoption of the technology has soared with more than $1 billion currently being spent annually by federal governments on CRISPR-based research [2]. However, while the CRISPR/Cas9 system has experienced rapid adoption, drawbacks of the system have become evident. CRISPR/Cas9 systems utilize either nonhomologous end-joining or homology-directed repair to restore DNA viability, both of which involve the repair of double-stranded DNA breaks [3]. In selecting a system that requires double-strand breaks, the prevalence of undesired insertions and/or deletions increases. Prime editing serves to overcome many of the limitations of the current CRISPR systems by allowing for genome manipulation using only single-strand DNA breaks (Figure 1) and may potentially give rise to a new frontier in genome editing research.

### 2.1. What Is Prime Editing?

Prime editing is a versatile genome editing method that “writes” new genetic information into precise DNA locations. This method differs from CRISPR/Cas9 systems in that it employs a catalytically impaired Cas9 endonuclease fused to a reverse transcriptase that is programmed via prime editing guide RNA (pegRNA) [1]. pegRNA encodes both the desired edit and the target DNA site. The prime editing method enables DNA insertions, deletions, and substitutions without requiring double-strand DNA breaks or exogenous donor DNA templates. By avoiding the sporadic DNA repair associated with double-strand breaks, prime editing can improve the accuracy of gene editing *in vitro* and theoretically also *in vivo*.

Unfortunately, the *in vivo* efficacy of prime editing systems is yet to be adequately demonstrated. To achieve widespread *in vivo* use, effective delivery of the prime editing machinery is required. Lentiviral systems have been proposed as transporters for the base editor 3 (BE3) system as its large size would not fit within generic adenoviral systems [4]; however, viral delivery systems often lead to mutagenic and carcinogenic side effects [5]. Therefore, a nonviral delivery system is preferred for patient safety reasons. Unfortunately, transfection efficiency is often lower for such systems. Ultimately, the development of effective nonviral delivery systems for prime editors will likely require innovation and a thorough evaluation of delivery system requirements.

## 3. Requirements for Delivery of Prime Editing Technologies

Before directly identifying the requirements for a delivery vehicle for prime editing technologies, it should be acknowledged that the optimal delivery vehicle will likely change depending on the specific disease target. Unfortunately, the very advantage of broad applicability of prime editing for a plethora of diseases is a major disadvantage in selecting the proper delivery method. A single delivery approach will not enable the full-breadth adoption of prime editing for all therapies designed to treat known human pathogenic genetic variants. However, certain techniques that provide a reasonable mode of delivery to a broad subset of disease targets and their broad requirements will be discussed here.

First, one of the most important considerations of prime editing delivery systems is that they must be able to deliver the entire prime editing complex. The entire complex is composed of a prime editing protein containing an RNA-guided DNA-nicking domain (usually Cas9 nickase) fused to a reverse transcriptase (RT) domain and complexed with prime editing guide RNA (pegRNA). Essentially, both protein and large RNA strand must be delivered. In theory, these components can be either codelivered in the same carrier or transported in separate carriers. However, in practice, codelivery of active agents in the same nanocarrier has generally resulted in greater therapeutic efficacy [6], likely by limiting opportunities for errors during administration and transport. Therefore, it is highly recommended that the nanocarrier be capable of carrying both prime editing protein and RNA as a PE-pegRNA complex while preventing the two materials from detrimentally interacting.

Second, the delivery of the PE-pegRNA complex tends to be more difficult than that of small-molecule drugs. In general, proteins for drug delivery exhibit notoriously short circulatory half-lives, poor absorption and permeability profiles, and high rates of denaturation during transport [7]. RNA molecules are similar in that they are readily metabolized when exposed in the bloodstream, induce immune responses in many extracellular environments, and demonstrate low tissue penetrance [8]. In essence, an optimal delivery system must fully cloak both protein and RNA components from the bloodstream and tissue interactions until its arrival at the target cell.

Third, genome editing technologies require delivery vehicles that enable intracellular and intranuclear (or intramitochondrial) uptake in the target cell. While many gene delivery systems utilize viral vectors, nonviral delivery systems are often preferred because of engineered control over toxicity profiles. Nonviral vectors are more advantageous over viral vectors due to their biosafety associated with less immunotoxicity. Plasmid DNA, liposome-DNA complexes (lipoplexes), and polymer-DNA complexes are examples of commonly used nonviral vectors. However, transfection efficiency of nonviral delivery systems regularly plummets. Alterations to the formulation of nonviral delivery systems to improve solubility (e.g., PEGylation) often work counterproductively when intracellular entry is necessary [9]. Furthermore, while hydrophilic surfaces restrict interactions with bloodstream components, they also frequently inhibit interactions with target cells. A designed prime editing delivery system should be engineered with a specific mechanism for target cell penetration and likely a method for intracellular motility and organelle uptake.

Fourth, the delivery system should enable a path for regulatory approval and therefore cannot be designed with extreme complexities. Drug delivery scientists discreetly shy away from admitting that very few nanomedicine systems are currently available in the commercial market, despite the large growth of interest in the scientific field. Many strong nanomedicine candidates fail to achieve set regulation standards as a result of the inability to account for all the degradation products, lack of demonstrated enhanced efficacy, or the triggering of system-mediated toxicities [10]. In the long run, it is strongly recommended to opt for systems that exhibit robust semblance to current commercially available nanomedicine products.

## 4. Advantages and Disadvantages of Specific Nanomedicine Systems

**Figure 2:**
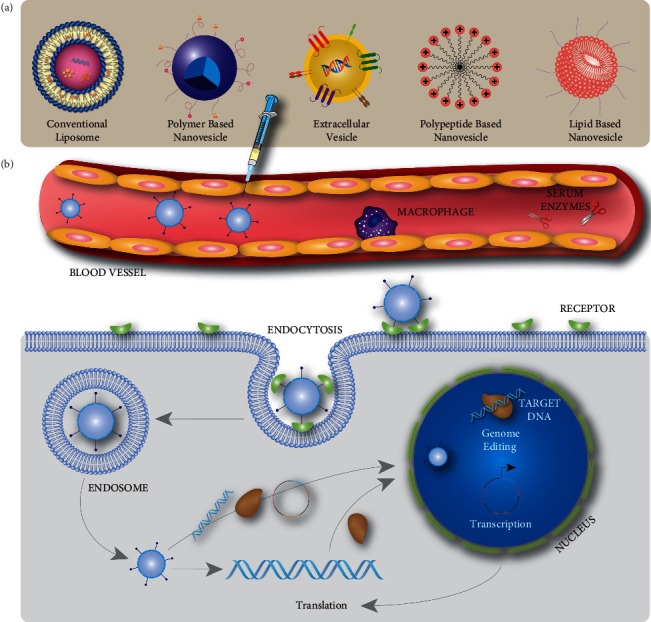
Illustration of nanovesicle types for CRISPR/Cas9 and single-guide RNA (sgRNA) delivery: (a) types of delivery systems for CRISPR/Cas9 and sgRNA and (b) genome-editing mechanisms of nanovesicle-delivered Cas9/sgRNA.

In general, a goal of nearly all drug delivery systems is to reduce off-target effects from the wide biodistribution of active pharmaceutical ingredients (APIs). Many factors need to be considered when designing an effective drug delivery system. Although it is beyond the scope of this article to fully list them here, some obvious considerations when engineering a system are to be cognizant of the nature of the drug (small molecule, large molecule, biologic, gene therapy, etc.), the particular tissues or cells being targeted, the drug modes of action, and the route of drug administration, as well as several other pharmacological factors. Despite the need for such an effort, the payoff can be monumental as the advantages of tailored drug delivery systems far outweigh nontargeted therapeutics. Even though local drug administration may aid prime editing delivery, local administration routes are not discussed here as systemic transporters likely have the broadest applicability for diseases susceptible to prime editing treatment. Accordingly, several nanotechnology-based delivery systems are addressed (Figure 2), and their potential for prime editing delivery (Table 1) is discussed.

### 4.1. Liposomes

Liposomes are vesicles composed of at least one lipid bilayer [11]. They are generally spherical, yet can assume other shapes with proper engineering. The bilayer structure of liposomes effectively serves as a barrier between the internal components and external surrounding fluid, allowing therapeutic agents to be protected during transport. The phospholipid assembly also enables hydrophilicity on both sides of the membrane, allowing the loading of water-soluble drugs within the liposome interior and the loading of lipophilic compounds by housing them within the bilayer. Drugs exhibiting an intermediate partition coefficient (logP) can segregate between the two phases. The liposomal compartmental space can house smaller liposomes, enabling unique architectures and the development of multilamellar liposome types. Specific lipids and other components can be tailored to increase the rigidity and stability of the liposomes or to ensure a slow-release vessel. Furthermore, mechanisms for targeting and tracking the vesicles can be incorporated within the liposomes throughout the majority of the synthesis process. Accordingly, liposomes should undoubtedly be considered when contemplating a straightforward nano-enabled delivery approach for new commercially relevant therapeutics.

With regard to the delivery of prime editing machinery, liposomes have several distinct advantages. First, the size of the liposomes can be optimized for cargo delivery, and the interior compartment space can house both prime editing protein and pegRNA. In fact, delivery of large protein-RNA complexes has been previously demonstrated using liposomes [12]. Second, several liposomal products have passed the US Food and Drug Administration (FDA) regulations and are commercially available. Adapting these currently approved liposomes for prime editing delivery would allow for a more direct path through the regulatory process. Finally, more advanced liposomal gene delivery systems have been engineered to enhance cellular [13], nuclear [14], and mitochondrial uptake [15] and could be examined for design innovation purposes.

However, liposomal systems often have several drawbacks. While liposomes mimic natural membranes, they are still foreign materials in the body and are known to be cleared by the mononuclear phagocytic system [16]. Efforts to use synthetic phospholipids and incorporate polyethylene glycol (PEG) coatings have somewhat lengthened the time to full blood clearance [17]; however, there are concerns accompanying these techniques regarding their ability to inhibit bloodstream extravasation. In addition, liposomal stability is a concern. Phospholipids sporadically jump from one membrane to another, leading to the occasional merging and coalescence of vesicles. Liposomes are not different and may often fuse membranes with unintended cells or bursts when in close proximity to other membranes. One estimate is that up to 30% of liposomal contents can be leaked in this manner, leading to large amounts of nontargeted API exposure [18]. *In vivo* fate of liposomes can be significantly affected by the interaction between liposomes and cells where they can be absorbed or undergo endocytosis. Stability during storage also remains an issue with most liposomal formulations requiring frozen storage conditions. If a specific prime editing therapy could initiate severe side effects without directed localization, liposomes may not provide the stability needed to adequately reduce off-target effects. Nonetheless, liposomes are likely to be strong candidate carriers for low-toxicity prime editing therapies.

### 4.2. Micelles

Micelles are the fundamental building blocks of emulsion-based formulations. A micelle is a three-dimensional assembly involving multiple amphiphilic surfactants. The hydrophobic ends (tails) of the multiple surfactant molecules arrange themselves near one another in order to minimize contact with water molecules, leading to a structure in which the hydrophilic ends encounter the water molecules at the periphery. Like liposomes, micelles are generally spherical, yet rod and planar structures can be obtained using surfactants with proper head-to-tail volume ratios and distinct solution conditions. By exchanging the solvent, inverse micelles can also be formed where the hydrophilic ends cluster and the hydrophobic ends interact with the solvent. Pharmaceutical formulations take advantage of both micelle types in the form of water-in-oil (W/O) emulsions and oil-in-water (O/W) emulsions. Advanced water-in-oil-in-water (W/O/W) and oil-in-water-in-oil (O/W/O) emulsions have also been developed to inhibit globular coalescence. Micelles carry molecular cargo by thermodynamically stabilizing the molecules in the core. Generally, fat-soluble agents are poorly soluble in surrounding solvents, leading to micelles being major applications in the transport of fat-soluble nutrients and drugs. Because of the simplicity of the system and the long history of using detergents, micelle systems are not always recognized as nanoparticle delivery systems, yet they have been shown to be effective in the delivery of genes and specific biologics [19]. For example, a recent study has demonstrated the potential of polyplex micelles in delivering Cas9 mRNA and guide RNA for *in vivo* genome editing in the mouse brain [20].

However, with regard to their potential for prime editing delivery, micelles face many challenges. First, micelles generally exhibit a size limitation, usually ranging from 2 to 20 nm, which can hinder the delivery applicability of larger macromolecules. In fact, amphiphilic block copolymers often self-assemble into micellar structures themselves [21], but rarely surpass the size limit as a stable structure. This inherent size limitation strongly negates the potential use of micelles to deliver protein-RNA complexes. Second, the stability of a three-dimensional supramolecular structure is difficult to maintain without covalent crosslinking. As such, internal contents are often “spilled” from micelles during delivery [22]. Third, micelles can activate the immune system and trigger rapid clearance. While PEGylation can decrease clearance, the presence of a hydrophilic chain can disrupt micellar stability, even in the case of amphiphilic polymeric micelles. Finally, as certain adverse reactions have been associated with strong surfactants, toxicity must always be considered when designing micelle-based systems. For these reasons, micelles are not recommended for the formulation of genome prime editing technologies.

### 4.3. Exosomes

Exosomes are similar in structure to liposomes, being membrane-bound vesicles; however, one intriguing difference sets them apart. Exosomes are derived from the endosomal compartment of cells and carry unique cell biomarkers that are characteristic of the cell of origin. Therefore, exosomes often display inherent targeting molecules on the outer surface of the membrane, enabling improvement in target cell uptake [23]. Exosomes are known to undergo endocytosis or fusion with the plasma membrane of target cells [24, 25], and internalized exosomes are degraded after delivering the cargo into the cytosol [25].

Exosomes can be engineered in a variety of sizes, and various exosome-loading procedures have been developed [26]. Recently, exosomes showed the potential in delivering prime editing proteins and gRNA. A recent study demonstrated that CIRSPR/Cas9 protein and sgRNA can be packaged into exosomes that in turn successfully transduced cells *in vitro* [27]. Nanomedic, an exosome-based CRISPR/Cas9 delivery system, showed promising efficiency of gene editing in various hard-to-transfer cell types, including human iPS cells, neurons, and myoblasts [28].

While it may initially seem that exosomes are an obvious choice for prime editing delivery over liposomes, certain drawbacks limit their utility, although not entirely. First, exosome engineering remains in its infancy, and the ability to design an exosome with firm-targeting capabilities is still somewhat only theoretical. Furthermore, alterations to exosomes after synthesis, for purposes such as bloodstream cloaking, tend to result in loss of desired exosomal properties [29]. Finally, the key biomarkers present in exosomes often lead to robust cytotoxicity, immune responses, and direct uptake by the reticuloendothelial system.

### 4.4. Polymer-Based Systems

Polymers are long-chain molecules composed of many repeating subunits (monomers). Theoretically, polymers assume a three-dimensional architecture in solution owing to interactions with the solvent environment. Hydrophilic polymers are commonly employed to improve the solubility of drugs with low solubility, while hydrophobic polymer components are often used for drug stabilization. Many different polymer types exist and can enable a wide range of properties relevant to drug delivery, namely, polymers can be covalently attached to drugs or utilize noncovalent interactions for drug transport. Furthermore, polymer nanosystems can be designed with various architectures, sizes, and compositions. As such, many different systems can be engineered to fully encapsulate both prime editing protein and pegRNA. Additionally, polymers enable facile attachment of *in vivo* tracking moieties, targeting molecules, and environmentally responsive entities and are thus often considered the most versatile platform for drug delivery. Upon arrival to the target cells, the most of polymer-based delivery vehicle undergo biodegradation or breakdown to initiate the delivery cargo into the cells.

While a polymer-based system may be a strong candidate for prime editing delivery, several factors should be considered. First, covalent attachment of a polymer to a protein may lead to incapacitation of the protein [30], and nearly all covalent attachments of polymers to RNA lead to loss of function. This being noted, polymer encapsulation is likely to be the best approach for prime editing delivery. It is not directly relevant to discuss all polymer types in this article; however, it should be mentioned that certain polymer types may reduce bloodstream interactions and/or immune responses to the nanosystem. For regulatory purposes, the determination of *in vivo* polymer degradation and excretion profiles is essential. Most regulatory agencies tend to look more favorably on liposomal systems than polymer-based systems, yet a few polymer-based systems have achieved US FDA approval [31]. However, any additional modifications to the polymer delivery system (e.g., to enhance tracking, transport, and targeting) will steepen the climb for regulatory approval. For research and grant-obtaining purposes, polymer-based systems can likely produce the best results, but from a commercialization standpoint, polymer nanosystems might introduce too many hurdles to merit their investigation.

### 4.5. Dendrimers

One of the most unique nanoparticle systems is the use of dendrimers, which are macromolecules composed of repeatedly branched chains. Their divergent synthesis begins with a single molecule at the core that then branches in multiple directions. Four branches become eight in the next stage of synthesis (called a generation), then sixteen, thirty-two, and so on. The dendrimer structure is unique in that the entire structure exhibits polymeric flexibility, yet the dendrimer surface exhibits an extremely high charge density. Dendrimers are known to chelate small molecules and ions within their branched structures, and their high charge density permits penetration across difficult *in vivo* barriers, such as the blood-brain barrier [32].

Dendrimers face similar issues as those of micelles when being evaluated for prime editing delivery. In theory, no dendrimer size limit should exist; however, in practice, it is very difficult to produce dendrimers beyond six generations. Quite simply, the charge density becomes too large, and steric repulsion forces restrict further branching. Therefore, the probability of developing a dendrimer system with sufficient cargo space to deliver both prime editing protein and pegRNA molecule is quite small.

### 4.6. Rigid Nanoparticle Systems

In this article, rigid nanoparticles are defined as nanoparticles composed of any material (organic, inorganic, metallic, etc.) that elicits a rigid morphology, including silica, metal oxide, and palladium nanoparticles. Essentially, rigid nanoparticle systems utilize conventional nanoparticles as delivery vessels. These systems can be designed using either top-down or bottom-up approaches. Top-down techniques tend to result in samples with high polydispersity in size and shape, whereas bottom-up methods can produce monodisperse samples with unique architectures. While surfaces can be chemically modified for the attachment of both proteins and nucleic acids, the attached molecules are on the surface of the particles and therefore presented to bloodstream components and rapidly disabled or cleared. Many of these systems rely solely on the adsorption of the APIs without chemical modification. However, these systems are likely to fail as prime editing delivery systems for the same reason.

On the contrary, certain rigid nanoparticle systems, such as silica, can be produced with a hollow core using bottom-up approaches, allowing for the encapsulation of various molecules [33]. As a result of the required etching processes, these hollow nanoparticles have a porous shell. Loading of proteins and nucleic acids into these particles could prove difficult depending on the pore size. Furthermore, if the pore size is too large, the molecular cargo will not be retained within the hollow core during transport. Methods have been developed to place an external shell around the hollow rigid nanoparticles to block pores and fully encapsulate drugs [34]; however, a nanoparticle system of such complexity has never attained federal regulatory approval. In addition, rigid nanoparticle systems often undergo surface dissolution to some extent in aqueous environments, leading to the release of various ions. In many cases, particularly with metallic nanoparticles, released ions pose considerable cytotoxic threats [35]. Overall, rigid nanoparticle systems should be avoided for prime editing delivery unless a strong rationale justifies their use.

## 5. Conclusion

Prime editing technologies have the potential to alter the genome editing space and achieve biomedical treatment breakthroughs. To realize this potential, proper delivery systems must be engineered for prime editing transport and localization. Prime editing delivery systems must be able to costabilize both prime editing protein and pegRNA during transport, possess a method for cellular internalization, and maintain a straightforward path to regulatory approval. Based on these criteria, liposomes are likely to be the most promising nanomedicine candidates for prime editing delivery if their potential toxicity and instability in the circulation system are well addressed. Polymer-based carriers with lower toxicity and higher stability may represent the second best option. However, the efficiency and safety of each delivery system must be carefully considered with the inevitable variance in between systems and cell types.

## Figures and Tables

**Table 1:** Nanomedicine systems for prime editing delivery.

Carrier	Advantages	Disadvantages
Liposomes	Versatile size control	Potential cytotoxicity
	Easy to design and engineer	Liposomal instability
	FDA clearance	Phagocytic clearance
Micelles	System simplicity	Lack of firm-targeting capability
	Cargo stability	Postsynthesis alteration may lose desired properties
		Innate biomarkers leading to robust cytotoxicity

Exosomes		
Polymer-based systems	A wide range of properties	Potential incapacitation
	Easy to incorporate a specific targeting method	Loss of protein function
Dendrimers	Easy penetration across difficult *in vivo* barriers	Cargo loading limitation
Rigid nanoparticles	Various fabrication techniques	Limited cargo production
		Potential surface dissolution

## Data Availability

No data were used to support this study.
